# Author Correction: Large composite fermion effective mass at filling factor 5/2

**DOI:** 10.1038/s41467-023-44392-8

**Published:** 2023-12-18

**Authors:** M. Petrescu, Z. Berkson-Korenberg, Sujatha Vijayakrishnan, K. W. West, L. N. Pfeiffer, G. Gervais

**Affiliations:** 1 Department of Physics, McGill University, Montreal, Quebec H3A 2T8 Canada; 2 Department of Electrical Engineering, Princeton University, Princeton, NJ 08544 USA

**Keywords:** Two-dimensional materials, Quantum Hall

Correction to: *Nature Communications* 10.1038/s41467-023-42986-w, published online 9 November 2023

The original version of this Article contained an error in Fig. 4, where the legend referenced Ref. [26] instead of Ref. [27]. This has been corrected in both the PDF and HTML versions of the Article.

The original version of the Supplementary Information associated with this Article contained an error in line 1 of paragraph 3 on page 8, which incorrectly referred to Ref. 26 instead of Ref. [27]. The HTML has been updated to include a corrected version of the Supplementary Information.

### Supplementary information

Updated Supplementary Information

