# Supplementary material for: Large composite fermion effective mass at filling factor 5/2
Source: Nat Commun. 2023 Nov 9;14:7250. doi: 10.1038/s41467-023-42986-w (PMC10636205; doi:10.1038/s41467-023-42986-w)
Supplement: Supplementary file 1 — Supplementary Information [file 41467_2023_42986_MOESM1_ESM.pdf]

Supplementary Information: Large composite fermion effective mass at filling factor 5/2

November 9, 2023

1 Conductance Measurement

In the main text of the manuscript, Fig. 1 provides the temperature dependent magneto-conductance measurements in the second Landau level. The circuit used for this measurement is depicted by Fig. S1A and has slightly different circuit components than the one used for the time-dependent conductance measurements based on the 2D electron gas (2DEG) response to a square wave (*i.e.* on/off input DC bias), displayed in Fig. S1B.

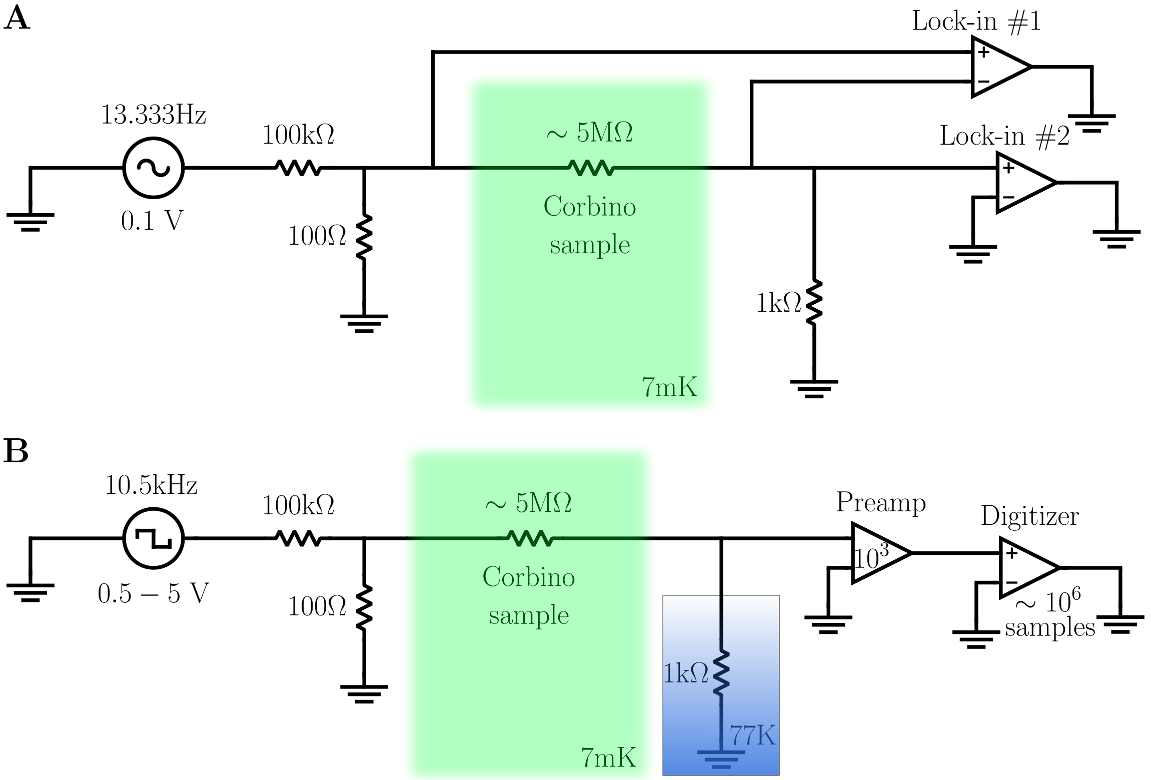

Figure S1: (**A**) Circuit used for basic two-point conductance measurement (transport). (**B**) Circuit used for specific heat measurements.

The main difference between the two circuits is the input signal. In Fig. S1A, a standard low-frequency sinusoidal wave is employed, whereas in Fig. S1B, a bipolar square wave of medium frequency is used. The main objective of the latter is to perform time-resolved measurements of the thermal time constant, τ. To achieve a better signal-to-noise ratio, a cold liquid nitrogen bath with a submerged 1 $k\Omega$ sensing resistor was added. Furthermore, we used a pre-amplifier with sufficient gain (1000) and a digitizer that averages over a million samples of signal.

There is significant improvement in the signal-to-noise ratio as the number of averages increases, however, at the cost of a drastically increased acquisition time. All the data acquired and presented in the main part of the manuscript was averaged in 100 batches for the lowest 2 biases (*i.e.* 0.5 V and 0.75 V) and in 50 batches for the other 17 biases (*i.e.* 1.0 V, 1.25 V, 1.5 V, ..., 4.75 V, 5.0 V). It is worth noting that the voltage divider (100 $k\Omega$ || 100 Ω) lowered the source bias by three orders of magnitude, thus lowering the actual input voltages for the Corbino ring 2DEG to 0.5 mV, 0.75 mV, 1.0 mV, ..., 4.75 mV, 5.0 mV. We used these input voltage values throughout the manuscript, for consistency.

2 Energy Gap Extraction from Arrhenius Fitted Conductance

Fig. S2 shows the conductance G of the 5/2 fractional quantum Hall state (FQHS) in an Arrhenius plot. The energy gap Δ is obtained by fitting the linear region of the conductance with respect to the inverse of temperature, *i.e* in the region where the electronic transport is activated, using $G = G_0 \left(e^{\frac{-\Delta}{2k_B T}} \right)$ where k_B is the Boltzmann constant. Please note the deviation from the linear regime for the lowest temperature data points is due to the transport becoming dominated by variable range hopping.

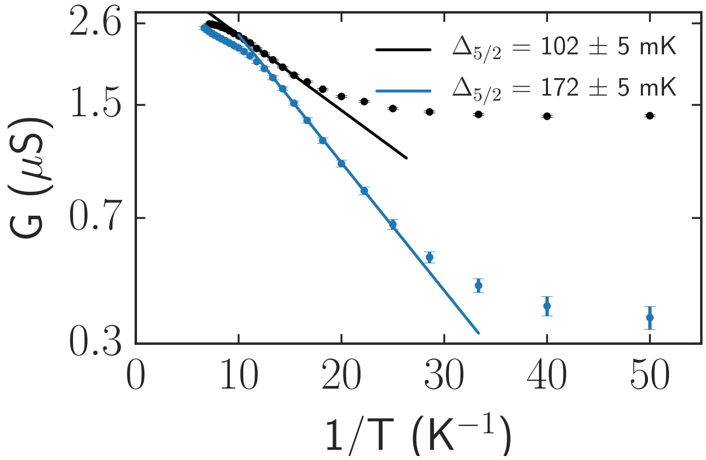

Figure S2: Conductance *versus* inverse of temperature (Arrhenius plot) for the 5/2 FQHS at exact filling value ($\nu^* = 0$). Previously reported energy gaps [31] are shown in black, whereas new measurements are shown in blue. The larger energy gap values reported now are due to both improvement in cooldown procedure and 2DEG preparation with a red LED, as well as improvements in the thermalization of electrons at the lowest temperatures. The reported error (blue and black error bars) shows the uncertainty in the conductance measurement.

3 *In Situ* Thermometer Calibration

We used the temperature dependence of conductance of the 5/2 FQHS to obtain a calibration for the electron temperature T_e. The conductance *versus* temperature dependence weakened as we moved away in magnetic field from each FQHS minima (*i.e.* away from exact filling factor, $\nu^* = 0$) towards a non-exact filling factor, $\nu^* \neq 0$. In order to extract the electron temperature T_e, a simple univariate spline interpolation was used for each data set. The temperature dependence below 20 mK, is very weak, although in Fig. 1 of the main text, the magneto-conductance of FQH states vary weakly down to below 10 mK. We can assess with certainty that an electron temperature of ∼20 mK was reached, and for this reason we only present data at $T_e \geq 20$ mK throughout the manuscript.

4 Shift and Subtract Method

The *shift and subtract* method was used to eliminate any unwanted transients due to wire resonance. Fig. S3 illustrates step-by-step exactly how the signal measured was converted to conductance using this method. The thermal relaxation time constant τ was obtained by fitting an exponential decay curve to the transient response of the 2DEG system, as shown in Fig. S3D. Here, temperature dependence of conductance was used as a thermometer (more details in the next subsection) in order to determine electron temperature as a function of the power applied, allowing for the extraction of thermal conductance K.

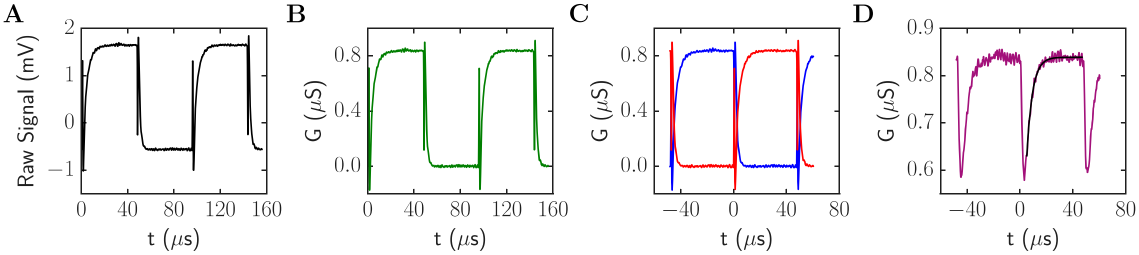

Figure S3: Example of time-resolved measurements at $\nu = 5/2$ during a square wave excitation with amplitude 2.5 mV at base temperature (7 mK). (**A**) Raw response of the 2DEG measured by the digitizer. (**B**) Conductance of the Corbino sample calculated with the measured current as well as the voltage drop across the sample. (**C**) The conductance G is shown in blue, along with the conductance offsetted by a half-period in red. Upon combination, the wiring resonance is subtracted out and the resulting conductance is shown in (**D**). An exponential decay fit (shown by a black line) was used to determine the relaxation time and the final conductance. In this example, their values are $\tau = 4.3 \pm 0.1$ μs and $G = 0.83 \pm 0.01$ μS, respectively.

5 Thermal Relaxation - Time Constant

The thermal relaxation time constant, τ, was obtained by performing the shift-and-subtract method described in Fig. S3, and then fitting to an exponential decay function,

$$G(t) = G_{eq} - \alpha e^{-t/\tau}, \tag{1}$$

where G_{eq} is the conductance at thermal equilibrium, t is time and α is the difference between G_{eq} and the conductance at $t = 0$, $G(0)$. In Fig. S4, we show the time dependent conductance measurements for all 19 input biases at a fixed thermal bath temperature. The two relevant parameters are conductance G and thermal time constant τ. They are both dependent on the input bias, V_{in}. In order to avoid any remnants of wire resonance, we opted to fit the conductance at $t \geq 5.2$ μs as shown in Fig. S4.

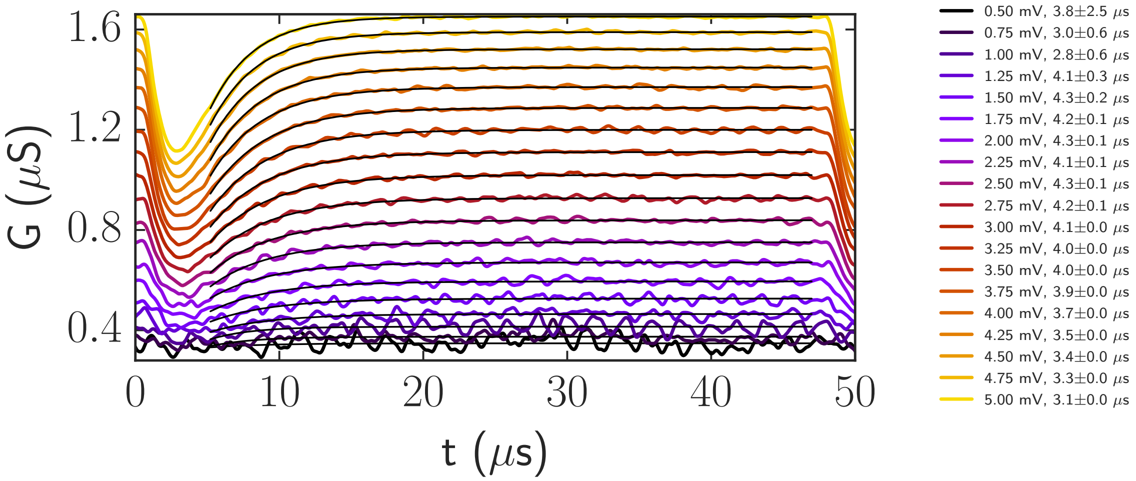

Figure S4: Conductance *versus* time for different input voltages at base temperature (*i.e.* 7 mK) for the 5/2 FQHS at $\nu^* = 0$. Each fit (shown in black) is used to extract τ. The data range used for each fit corresponds to the length of each corresponding black curve (*i.e.* $t \in [5.2, 47.0]$ μs).

6 Thermal Conductance

For each measurement, the power $P = GV_{in}^2$ was computed since the DC thermal conductance depends on the ratio of power to temperature difference ΔT_e as well as system size (*i.e.* the area, $\mathcal{A}$). In the fitting process, we payed careful attention to the interval over which the fit was performed. We kept the same interval range of input biases as our previous work [31], however the scale was refined by roughly doubling the number of different input biases used (from 10 to 19).

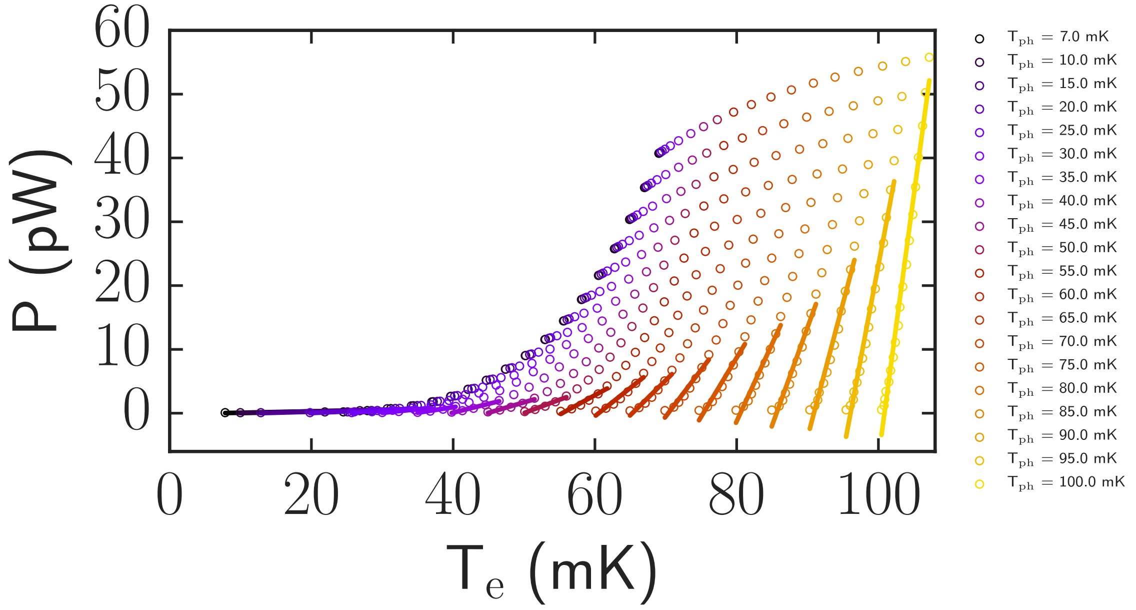

Figure S5: Power *versus* temperature for the 5/2 FQHS at $\nu^* = 0$. The slope of each linear fit is proportional to K.

The constraints used in [31] were maintained, *i.e.* fitting a minimum of 4 points if the temperature interval spanned by them was larger or equal to 7 mK. As mentioned in [31], we used the low-power limit to extract K since the temperature dependence of P is non-linear for large ΔT_e intervals, especially for data at lower thermal bath temperatures T_{ph}, see Fig. S5.

7 Temperature Dependence of Specific Heat

In Fig. S6, we present the specific heat temperature dependence for all data sets used in the main manuscript final results (Fig. 4). The top panels illustrate data reproducibility at exact effective filling factor $\nu^* = 0$.

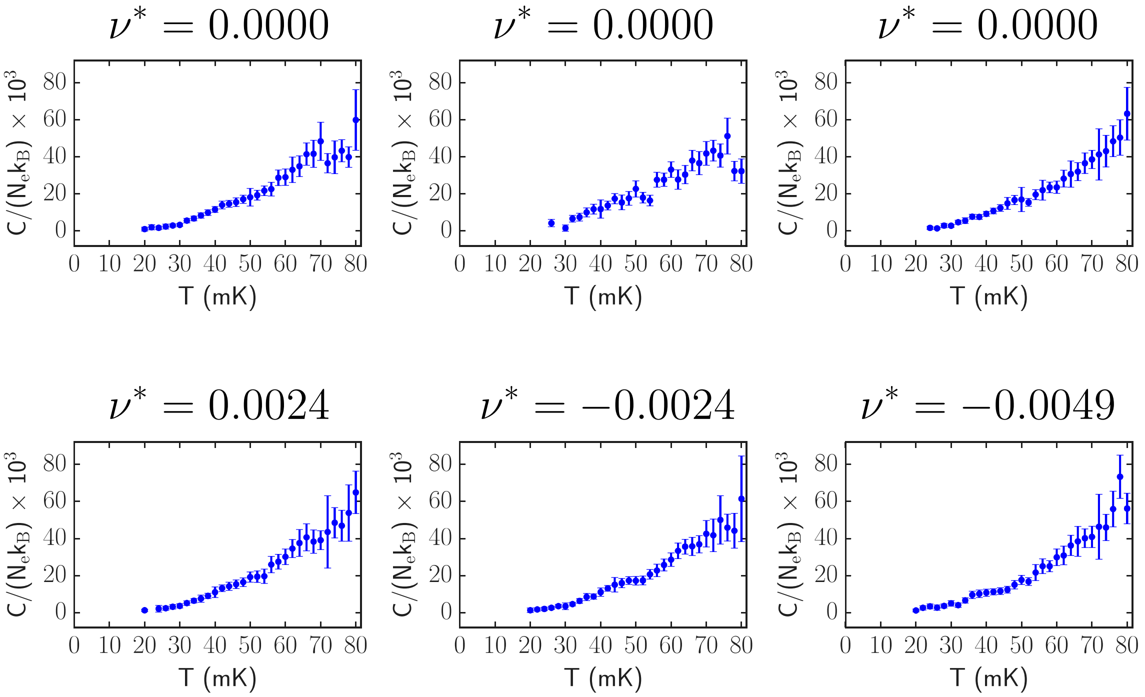

Figure S6: Specific heat per electron per k_B *versus* temperature for different data sets at exact effective filling factor $\nu^* = 0$ on the top row and at non-exact effective filling factor $\nu^* \neq 0$ on the bottom row. The error bars for the specific heat are showing the statistical errors propagated from the measurements of τ and K, see section 9 of this supplementary information document.

8 Specific Heat in Higher Landau Levels

A control experiment was carried out in the same Corbino sample in higher Landau levels. The results of the experiment are shown in the Figure S6 of the supplementary material of Ref. *Phys. Rev. B* **95**, 201306 (2017) published by our group. As expected, the measured specific heat follows the conductance and hence the density of states near the Fermi energy. Owing to the very high quality of the Corbino sample used, weak electronic bubble phases could be observed in the flanks of the conductance at integer fillings, and these were also observed within some degree in the specific heat measurements.

9 Uncertainty/Error

The main source of uncertainty in the specific heat data ($C = \tau K$) originates from its constituents, *i.e.* the thermal relaxation time constant τ and the thermal conductance K. A fully detailed overview can be found in Ref. [30-32]. Throughout the manuscript, the statistical error from the measurements was propagated each time an algebraic operation was performed. As can be seen in Fig. 2 and Fig. 3 of the main text, the errors were also propagated to the specific heat over temperature ratio (C/N_eT). This error was then compared with other sources of errors present in our experiment/analysis, such as *in situ* thermometry uncertainty along with the corresponding propagated uncertainty in τ, K and C/N_eT.

The thermopower entropy data was determined using a digitized version of the data of Ref. [27], with uncertainty determined by visual inspection of the local noise, and overall background fluctuations.

The effective mass ratio m^*/m_e estimated from the limiting value of C/N_eT and entropy considerations has multiple sources of uncertainty. However, we have chosen to quote m^*/m_e using the limiting value method as an estimate, as is shown in the Fig. 4 of the main manuscript. Since these values are estimated based on entropy conservation and the assumption of a CF Fermi liquid at temperatures above the many-body energy gap, we opted to present only the centroid of the estimate. In any case, all methods that we considered to estimate the effective mass led to m^*/m_e being considerably larger than one, and roughly two to three times larger than in the first Landau level at 1/2 filling factor, provided that the impurity scattering parameter p_{CF} does not greatly differ than in the first Landau level (see main text).

10 Reproducibility

Consistency tests were also performed within the same cool-down but on different days (*i.e.* datasets) at the same relative filling factor ν^*. Fig. S7 shows the comparison of two different datasets for 5/2 FQHS at exact filling factor value $\nu^* = 0$.

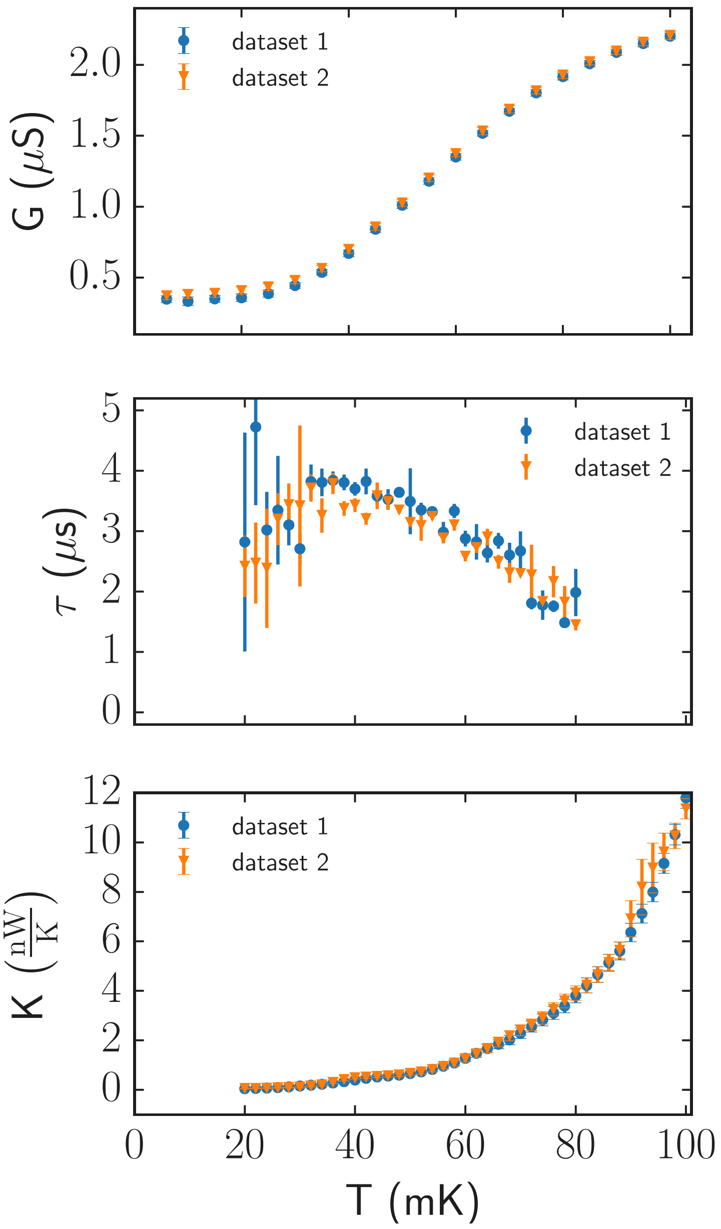

Figure S7: Conductance (top panel), thermalization time constant (middle panel) and thermal conductance (bottom panel) *versus* temperature for 5/2 FQHS at $\nu^* = 0$ for two different datasets. The error was determined as described in section 9 of this supplementary information document.

All the specific heat measurements shown above were acquired in a cryostat (a "dry" Bluefors BD-250) different than in our previous work performed in a "wet" Janis JDR-150 [31]. Much differs between the two, with the main difference being the overall distance between room and base temperature, and hence the wiring length, with the end effect of generating much distinct wire resonances. In both cases, the experiment could be performed and benchmarked against. Importantly, improvements in the thermalization and cooling procedure in this current work allowed us to significantly increase the FQH gap value, to optimize the electron cooling, to lower the electron temperature reached, and confirm the low-temperature behaviour of the specific heat below 40 mK, which had not been possible previously in Ref. [31].
